# First Case of *KRT2* Epidermolytic Nevus and Novel Clinical and Genetic Findings in 26 Italian Patients with Keratinopathic Ichthyoses

**DOI:** 10.3390/ijms21207707

**Published:** 2020-10-18

**Authors:** Andrea Diociaiuti, Daniele Castiglia, Marialuisa Corbeddu, Roberta Rotunno, Sabrina Rossi, Elisa Pisaneschi, Claudia Cesario, Angelo Giuseppe Condorelli, Giovanna Zambruno, May El Hachem

**Affiliations:** 1 Dermatology Unit, Bambino Gesù Children’s Hospital, IRCCS, Piazza S. Onofrio, 4, 00165 Rome, Italy; marialuisa.corbeddu@opbg.net (M.C.); roberta.rotunno@opbg.net (R.R.); may.elhachem@opbg.net (M.E.H.); 2 IDI-IRCCS, Via dei Monti di Creta, 104, 00167 Rome, Italy; d.castiglia@idi.it; 3 Pathology Unit, Bambino Gesù Children’s Hospital, IRCCS, Piazza S. Onofrio, 4, 00165 Rome, Italy; sabrina2.rossi@opbg.net; 4 Medical Genetics Laboratory, Bambino Gesù Children’s Hospital, IRCCS, Piazza S. Onofrio, 4, 00165 Rome, Italy; elisa.pisaneschi@opbg.net (E.P.); claudia.cesario@opbg.net (C.C.); 5 Genetics and Rare Diseases Research Division, Bambino Gesù Children’s Hospital, IRCCS, Piazza S. Onofrio, 4, 00165 Rome, Italy; agiuseppe.condorelli@opbg.net (A.G.C.); giovanna.zambruno@opbg.net (G.Z.)

**Keywords:** KRT1, KRT10, KRT2, keratin, epidermolytic ichthyosis, superficial epidermolytic ichthyosis, ichthyosis with confetti, epidermal nevus, histopathology, ultrastructure

## Abstract

Keratinopathic ichthyoses (KI) are a clinically heterogeneous group of keratinization disorders due to mutations in *KRT1*, *KTR10*, or *KRT2* genes encoding keratins of suprabasal epidermis. Characteristic clinical features include superficial blisters and erosions in infancy and progressive development of hyperkeratosis. Histopathology shows epidermolytic hyperkeratosis. We describe the clinical, histopathological, and molecular findings of a series of 26 Italian patients from 19 unrelated families affected with (i) epidermolytic ichthyosis due to *KRT1* or *KRT10* mutations (7 and 9 cases, respectively); (ii) *KTR10*-mutated ichthyosis with confetti (2 cases); (iii) *KRT2*-mutated superficial epidermolytic ichthyosis (5 cases); and (iv) *KRT10*-mutated epidermolytic nevus (2 cases). Of note, molecular genetic testing in a third case of extensive epidermolytic nevus revealed a somatic missense mutation (p.Asn186Asp) in the *KRT2* gene, detected in DNA from lesional skin at an allelic frequency of 25% and, at very low frequency (1.5%), also in blood. Finally, we report three novel dominant mutations, including a frameshift mutation altering the C-terminal V2 domain of keratin 1 in three familiar cases presenting a mild phenotype. Overall, our findings expand the phenotypic and molecular spectrum of KI and show for the first time that epidermolytic nevus can be due to somatic *KRT2* mutation.

## 1. Introduction

Keratinopathic ichthyoses (KI) are a group of genetic skin diseases due to mutations in keratin genes *KRT1*, *KRT2*, and *KRT10* encoding keratins 1, 2 and 10 (K1, K2, K10), respectively, expressed in suprabasal epidermis [1]. They have been included in classifications of both ichthyoses and skin fragility disorders due to the presence of hyperkeratosis as well as of trauma-induced superficial blisters and erosions [1,2]. In KI, epidermal fragility and blister formation following mechanical stress attest to the structural role of cytoskeleton keratins in epidermal homeostasis, while hyperkeratosis and scaling mainly result from epidermal hyperproliferation secondary to skin barrier defects [3]. KI comprise epidermolytic ichthyosis (EI, OMIM #113800), superficial epidermolytic ichthyosis (SEI, OMIM #146800), and congenital reticular ichthyosiform erythroderma (CRIE, OMIM #609165). In addition, a mosaic disease, epidermolytic nevus (EN), is included because of the possibility of gonadal mosaicism and, thus, offspring affected with EI. 

EI, previously known as epidermolytic hyperkeratosis or bullous congenital ichthyosiform erythroderma of Brocq, manifests at birth with widespread superficial erosions and blisters on erythrodermic skin. In the majority of cases, it requires hospitalization in a neonatal intensive care unit due to highly defective skin barrier function. Over time, blistering is superseded by progressive skin thickening, manifesting as adherent hyperkeratosis and verrucous malodorous lesions of variable severity. Some patients also develop palmoplantar keratoderma (PPK). EI is usually due to heterozygous missense mutations or small insertion or deletion mutations in *KRT1* or *KRT10* genes and inherited as an autosomal dominant trait, with about 50% of cases occurring de novo and with patients carrying *KRT1* mutations typically presenting severe PPK. However, very rare cases of recessively inherited EI due to homozygous mutations in *KRT10* have been described [4,5,6,7]. 

CRIE, also known as ichthyosis with confetti, is a peculiar non-blistering KI subtype caused by dominant negative mutations in *KRT10* or, less commonly, *KRT1* [8]. It is characterized clinically by the progressive development of small areas of normal-looking skin over a erythroderma background. These confetti-like skin spots represent “repaired” skin due to independent events of somatic reversion of keratin gene mutations via mitotic recombination.

SEI, previously known as ichthyosis bullosa of Siemens, presents a phenotype similar to EI, but with milder expression, which usually improves with age, and no PPK. It is caused by heterozygous mutations in the *KRT2* gene encoding K2, which is expressed in the uppermost epidermal layers. 

EN is characterized by hyperkeratotic verrucous streaks which are distributed along Blaschko lines and appear gradually after birth [9]. EN can be localized or have a unilateral or bilateral distribution. In the majority of cases, it is caused by post-zygotic mutations in the *KRT10* gene, while *KRT1* mutations have been detected in a few patients [10,11,12,13]. Of note, somatic mutations in EN can involve germline cells and can thus be transmitted to the offspring, causing EI. 

The diagnosis of KI is based on clinical features combined with histological and ultrastructural findings [1]. Histopathology reveals the typical features of hyperkeratosis with epidermolysis, i.e., vacuolar degeneration of suprabasal epidermal layers with coarse keratohyalin granules and eosinophilic inclusions. Transmission electron microscopy (TEM) examination allows the pathognomonic clumps of keratin intermediate filaments to be visualized, which represent the ultrastructural counterpart of eosinophilic inclusions seen by light microscopy in suprabasal epidermal layers. 

However, clinics and histopathology do not always allow EI to be easily distinguished from SEI, given the phenotypic heterogeneity of both disorders and their clinical and histopathological similarities [14]. Thus, genetic testing is relevant to establish the correct diagnosis and to offer appropriate genetic counselling. 

We describe the clinical, histopathological, and molecular features of a series of 26 patients from 19 unrelated families addressed to our center, including one patient presenting with EN in whom a *KRT2* mutation was identified somatically for the first time.

## 2. Results

The series consisted of 26 cases, 12 males and 14 females, from 19 families (Table 1). Twelve cases were sporadic and due to de novo mutations. In two families, three affected members, two siblings and one parent (*n*. 1–3 and 23–25 in Table 1), and in three families two members, a child and one parent (*n*. 5–6, 10–11, and 14–15), were examined. Fifteen patients were aged <18 years, and the remaining 11 were adults. Follow-up information was available in 21 patients. Histopathological and TEM examination were performed in 12 and 8 patients, respectively. Sixteen patients were affected with EI, five with SEI, three with EN, and two with CRIE. Onset was in the first days of life in 15 EI and in the two CRIE patients, and in the first to second year in SEI. Finally, one patient with EI reported that her disease was first noticed at 6 months (*n*. 3).

PPK was present in nine EI patients, corresponding to the seven cases with *KRT1* mutations and one case carrying a *KRT10* mutation, in the two CRIE patients, and in one SEI case (*n*. 22) which has been previously reported [15]. Interestingly, 15 cases, including the 2 CRIE, all 5 SEI, and 8 EI cases, manifested hypertrichosis, which was frequently more evident on the limbs. In particular, hypertrichosis was present in five EI patients with *KRT1* mutations and PPK (*n*. 1, 2, 5–7), as well as in three EI patients carrying *KRT10* mutations (*n*. 9, 13, 14), one also presenting PPK (*n*. 13). Hypohidrosis was a variable finding, being reported in eight patients (four EI, one SEI, two CRIE, one EN). Six cases (four EI, two CRIE) developed over time mild to severe limb flexion contractures, and growth delay was documented in six patients (four EI, two CRIE). Reported symptoms comprised itching in 16 cases (eight EI, five SEI, two CRIE, one EN), severe pain in the two CRIE, and occasional pain mainly at fissure sites in several other patients. 

**Figure 1:**
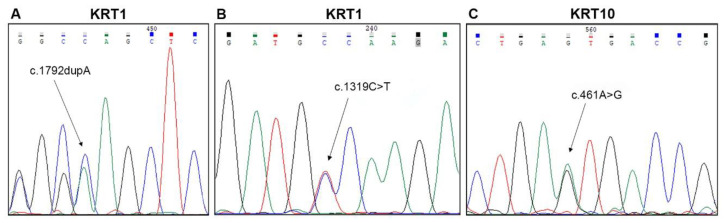
Molecular findings in three familial and two sporadic cases of epidermolytic ichthyosis. Sequence electropherograms showing the three novel mutations identified: (**A**) c.1792dupA (p.Ser598Lysfs*56), (**B**) c.1319C > T (p.Ala440Val) in *KRT1* (NM_006121) (patients *n*. 1–3 and 4 of Table 1, respectively), and (**C**) c.461A > G (p.Asn154Ser) in *KRT10* (NM_000421) (*n*. 13 of Table 1).

Molecular genetic testing was performed in 25 out of 26 KI patients of our series. We identified 3 novel and 10 previously described mutations, 6 of which were in *KRT10* (48% of the disease alleles, 12/25), 4 in *KRT1* (28%, 7/25), and 3 in *KRT2* (24%, 6/25) (Table 1, Figure 1 and Figure S1). The higher frequency of mutations detected in *KRT10* compared to the other KI genes is mostly due to the spontaneous occurrence of C-to-T transitions affecting the CG dinucleotide in the “hotspot” codon 156, which was mutated in seven patients (*n*. 8, 9, 12, 14–17), four of whom were sporadic EI cases (*n*. 8, 9, 12, 16) and two were presenting with mosaic EN (*n*. 15, 17).

**Figure 2:**
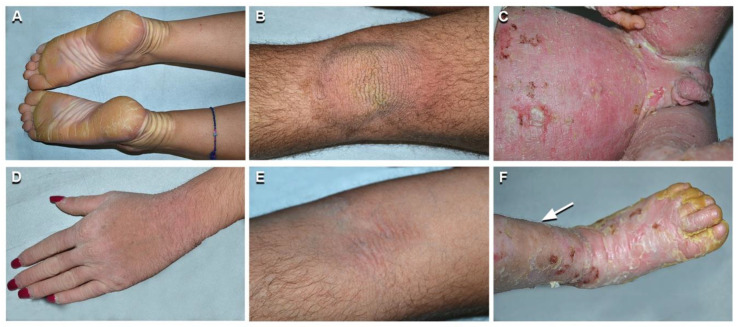
Phenotypic spectrum of epidermolytic ichthyosis (EI). Mild EI phenotype in two siblings (patients *n*. 1 and 2 of Table 1): yellowish plantar keratoderma with erythematous halo, left hand dorsum hyperkeratosis and hypertrichosis on the upper left limb in the sister aged 19 years (*n*. 2 of Table 1) (**A**,**D**); yellowish verrucous hyperkeratosis of the left knee and mild linear hyperkeratosis in the left antecubital fossa in the brother aged 21 (*n*. 1 of Table 1) (**B**,**E**). Severe and diffuse erythroderma, with erosions, crusts, and yellowish thick hyperkeratosis in a 3-year-old child (**C**,**F**) (*n*. 13 of Table 1). Note leg hypertrichosis in F (arrow).

Among cases with peculiar clinical features, patients *n*. 1 and 2 were siblings and presented an unusually mild phenotype, which had been diagnosed as lamellar ichthyosis until 21 years of age when they came to our observation. They reported disease onset at birth, but no overt blistering in infancy and childhood. In adulthood, both patients had diffuse PPK associated with hyperkeratosis localized on the lower back, elbows, knees, and dorsum of hands and feet, in the absence of skin fragility signs (Figure 2A,B,D). In addition, mild hyperkeratosis in a linear array was present on the axillary, antecubital, and popliteal folds (Figure 2E). Both siblings had limb hypertrichosis (Figure 2D) and face erythema. They were in good general health. Their mother (*n*. 3) also showed similar, though milder, ichthyosis features. The novel frameshift mutation, c.1792dupA, in *KRT1* (NM_006121.3) was identified at the heterozygous state in the two siblings and their mother (Figure 1A). The frameshift p.Ser598Lysfs*56 mutation is located in the V2 tail domain of K1 and results in a stop codon readthrough that leads to the insertion of seven additional amino acids at the C-terminus. Two previously identified frameshift mutations listed in the Intermediate Filament Mutation Database (http://www.interfil.org), p.Tyr587Leufs*67 and p.Gly585Trpfs*69, have similar consequences at the C-terminal end of K1 (Figure S2) and were associated with mild phenotypes of EI, as in our family [16,17].

Patient *n*. 4 was peculiar for her mild ichthyosis phenotype associated with diffuse and severe PPK. Disease onset was at 2 days of age with trauma-induced superficial erosions and desquamation of the ankles, axillary folds, and face. Skin fragility disappeared within one month. PPK and neck hyperkeratosis started to develop by 3 months of age. A skin biopsy and blood sampling for genetic analysis were obtained at 7 years, when the patient presented yellowish diffuse PPK with a sharp erythematous border on the wrists and foot lateral surface, associated with knee, elbow, and neck focal hyperkeratotic plaques. She was in general good health and complained only about wrist itching. Her father was affected by severe PPK. Histopathology showed orthokeratotic hyperkeratosis and focal keratinocyte vacuolization with coarse and irregular keratohyalin granules and eosinophilic cytoplasmic inclusions in the upper epidermal layers. TEM confirmed focal vacuolization in the suprabasal layers, together with tonofilament aggregation and clumping, predominantly in the granular layer. Genetic analysis identified the novel amino acid substitution c.1319C > T; p.Ala440Val in *KRT1* (NM_006121.4) at the heterozygous state in both the patient and her father (Figure 1B). A previously reported mutation located just upstream residue Ala440 (p.Leu437Pro) resulted in a pure PPK phenotype in a multigenerational family [18]. Such mild phenotypes are in line with the position of both mutations within the central part of the K1 2B domain, i.e., upstream the helix termination peptide where mutations impact more severely the keratin function. 

On the other hand, patient *n*. 13 presented a severe phenotype. He was born with erythroderma and generalized superficial blisters and erosions, requiring intensive care at the local hospital, where EI was diagnosed based on histopathological findings of a skin biopsy. The patient was addressed to us at three months of age when he presented diffuse whitish large scales with numerous erosions on erythrodermic skin. Over time, the scales became thicker, more adherent, and yellowish, involving also palmoplantar areas, while erosions affecting the entire skin surface persisted (Figure 2C,F). At the age of 8 years, he continues to present growth delay (weight and height < 3rd percentile) and has developed limb contractures. Genetic testing revealed the novel p.Asn154Ser mutation in domain 1A of K10 (Figure 1C). The EI phenotype of our child appears more severe than a previously reported adult case carrying a different amino acid substitution, p.Asn154His, at the same codon [19]. Asparagine154 is located within the heptad repeat of the α-helix 1A domain in a position that is usually hydrophilic. Asparagine and histidine have hydrophilic side groups, though His is bulkier and less flexible. A serine side group is in between that of a hydrophilic and hydrophobic amino acid, but it is smaller than His. Provean prediction (http://provean.jcvi.org/index.php) calculates an almost identical value of pathogenicity for both mutations (Asn154His, score −4.372; Asn154Ser, score −4.329; score threshold −2.5). Thus, it is difficult to predict whether the p.Asn154Ser is more deleterious than the p.Asn154His to explain the seemingly more severe phenotypic consequences of the former compared to the latter. 

Patient *n*. 14 was born with erythroderma and diffuse superficial blisters and erosions, requiring hospitalization. A skin biopsy was diagnostic for EI, and TEM documented extensive tonofilament clumping and cytolysis of suprabasal epidermis. In the following months, erythroderma and trauma-induced blistering decreased, while yellow-brownish adherent hyperkeratosis progressively developed all over the body with a cobblestone pattern on extensor surfaces. Palmoplantar surfaces and face were spared. The patient presented growth delay (weight and height < 3rd percentile), severe itching, hypohidrosis, hypertrichosis, and flexion contractures of the lower limbs. Blistering tendency worsened during summer in the first years of life, while hyperkeratosis continues to be more evident in wintertime at the age of 10 years. Interestingly, clinical examination of the mother revealed a systematized EN affecting the trunk and limbs bilaterally in a blaschkoid pattern. Genetic analysis identified the hotspot missense mutation c.466C > T; p.Arg156Cys in *KRT10* (NM_000421.3) at heterozygous state in the proband; the mutation was also detected with an allelic frequency of 5% in blood genomic DNA of the mother, in line with her extensive EN. 

**Figure 3:**
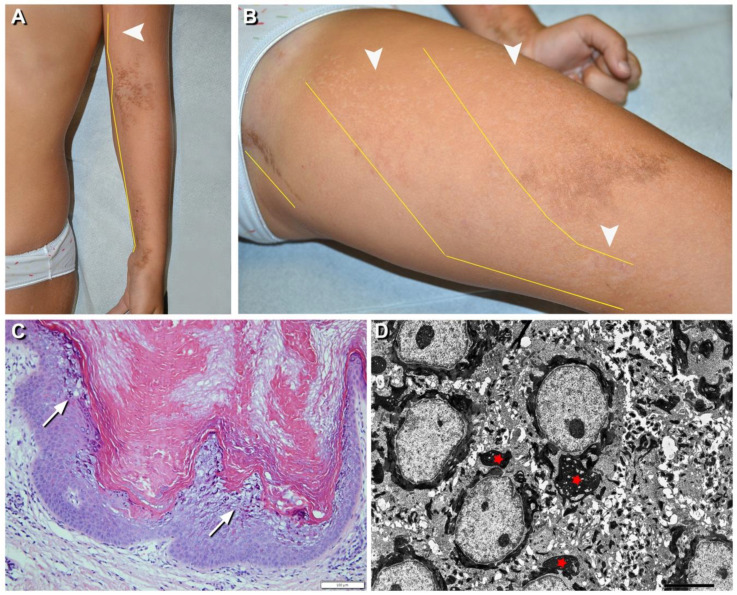
Clinical, histopathological, and ultrastructural findings in epidermolytic nevus (EN) due to *KRT10* somatic mutation. Hypopigmented (arrowheads) and brownish hyperkeratotic linear lesions following a blaschkoid distribution (yellow lines) on the left upper and lower limbs (**A**,**B**) of a 7-year-old girl (*n*. 17 of Table 1). Focal vacuolar degeneration (arrows) of suprabasal epidermal layers with hypergranulosis and compact hyperkeratosis. Numerous eosinophilic cytoplasmic inclusions in suprabasal epidermal layers are also visible (**C**) and correspond to perinuclear tonofilament aggregation and clumping (asterisks) visualized by ultrastructural examination (**D**). Bar: 100 µm in (**C**) and 5.0 µm in (**D**).

The second patient with EN (*n*. 17) had hypopigmented and brownish hyperkeratotic blaschkoid lesions limited to the left lower and upper limbs (Figure 3A,B). Histopathology of lesional skin showed typical features of EN with focal epidermolysis of the Malpighian and granular layers, irregular keratohyaline granules, and eosinophilic inclusions, and TEM confirmed the presence of tonofilament clumps in suprabasal keratinocytes (Figure 3C,D). In keeping with the limited extent of the EN, genetic analysis did not detect any sequence variant in genomic DNA from blood, while the recurrent missense *KRT10* mutation p.Arg156Cys was present, with an allelic frequency of 18%, in the DNA purified from the whole biopsy taken from lesional skin. Thus, in two (*n*. 15, 17) of the three EN patients present in our series, the hypermutable and vulnerable codon 156 of K10 was involved, as expected.

**Figure 4:**
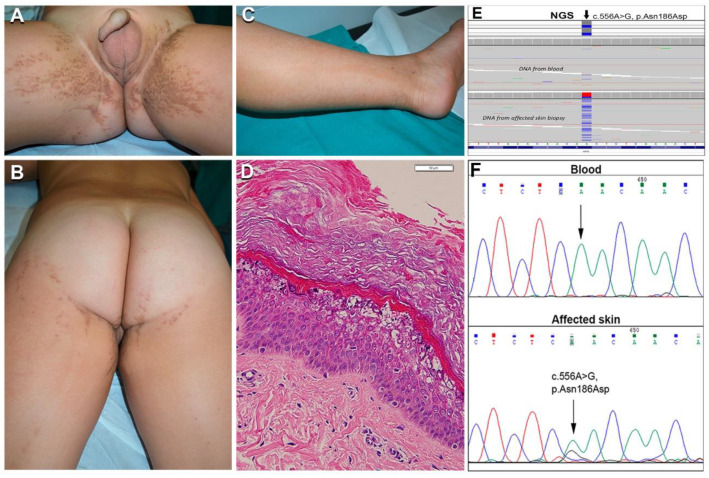
Clinical, histopathological, and molecular genetic findings in the first case of epidermolytic nevus (EN) due to *KRT2* somatic mutation. Extensive bilateral brownish hyperkeratosis and hypopigmentation, both following Blaschko lines, on the inguinal folds, gluteal and perineal regions, thighs, and left leg (**A**–**C**) in a boy aged 3 years (*n*. 26 of Table 1). Histopathology of a hypopigmented lesion shows vacuolar degeneration of suprabasal epidermal layers with irregular keratohyalin granules and eosinophilic cytoplasmic inclusions, in addition to hyperkeratosis (**D**); bar: 50 µm; similar findings accompanied by acanthosis and thicker hyperkeratosis were present in hyperkeratotic lesions (not shown). Mutant alleles with the *KRT2* c.556A > G somatic mutation were detected by NGS sequencing both in DNA from blood (estimated frequency 1.5%) and affected skin (25%) (**E**). Sanger sequencing failed to detect the mutation in blood DNA but confirmed it in the DNA from affected skin, as demonstrated by the lower peak of nucleotide G under the wild-type A (**F**).

The third patient with EN, a 7-year-old child (*n*. 26), presented linear blaschkoid brownish hyperkeratosis bilaterally, on lower and upper limbs, inguinal and infragluteal folds, as well as hypochromic streaks on the back and limbs (Figure 4A–C). The skin disorder had been noticed in the first year of life initially on the legs and had progressively spread to the other parts of the body, sparing the face and palmoplantar regions. Histopathological examination documented similar features of hyperkeratosis, namely focal vacuolar degeneration of upper epidermis, with coarse keratohyalin granules and eosinophilic inclusions in both hypochromic and hyperkeratotic lesions (Figure 4D).

Unexpectedly, genetic analysis of this patient revealed the missense mutation in the *KRT2* gene (NM_000423.2), c.556A > G; p.Asn186Asp, in DNA from lesional skin and blood (Figure 4E,F). Specifically, this pathogenic change was detected in 335 out of 1321 reads (25%) across the DNA purified from the whole biopsy of the affected skin, and in 18 out of 1186 reads (1.5%) in the DNA from blood; in contrast, a different non-pathogenic *KRT2* single nucleotide variant (p.Ser44Arg) was observed in heterozygosity in approximately 50% of the sequenced alleles from both skin and blood DNA, demonstrating it was inherited from the germline. The somatic mutation p.Asn186Asp detected in our patient is located in the K2 1A domain and has been reported earlier in the literature in a case with full-blown SEI [20]. To our knowledge, this is the first example of mosaicism due to a somatic mutation in *KRT2*.

The follow-up of the two previously described children with CRIE (*n*. 19, 20) [21] confirmed the progressive appearance and increase in size of the spots of normal looking skin as well as the severe hyperkeratosis, especially over joints and palmoplantar areas, causing flexion contractures of the limbs and serious functional damage with gait abnormality. Hypertrichosis was particularly prominent in these patients and affected not only the limbs but also the trunk. Moreover, patient *n*. 20 presented mental retardation. In these patients, white spots of normal looking skin were not investigated by genetic analysis [21].

**Figure 5:**
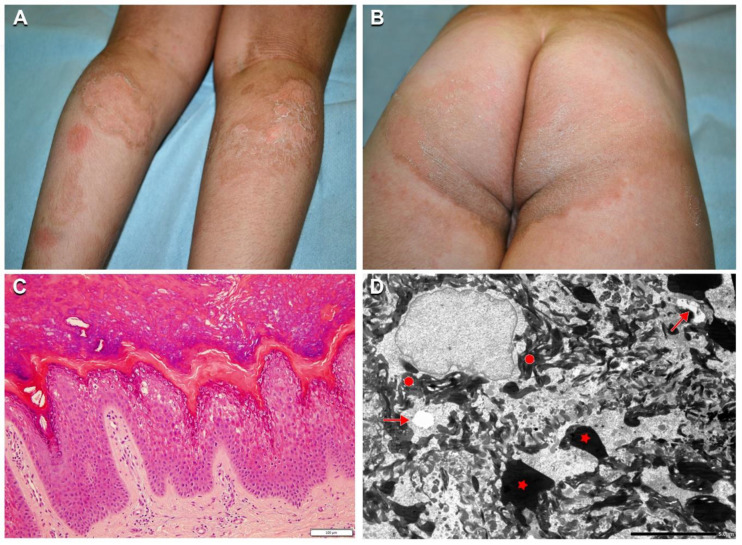
Clinical, histopathological, and ultrastructural findings in superficial epidermolytic ichthyosis (SEI). Brownish hyperkeratosis on the buttocks and infragluteal folds with linear hyperkeratosis on the right popliteal fossa in a 7-year-old boy (*n*. 23 of Table 1) (**A**,**B**). Note the two erythematous patches on the left calf, and polycyclic mildly erythematous and focally eroded areas with peripheral superficial lamellar desquamation on both calves (**A**). Leg hypertrichosis is also evident (**A**). Skin histopathology shows vacuolization of the granular and upper suprabasal epidermal layers, with numerous eosinophilic inclusions (**C**). Ultrastructural examination reveals initial cytoplasmic vacuolization in the granular layer (arrows) with extensive tonofilament clumps (filled circles) and keratohyalin granules of irregular size and shape (asterisks) (**D**). Bar: 100 µm in (**C**) and 5.0 µm in (**D**).

Two additional patients affected with SEI (*n*. 23, 24), carrying the known *KRT2* missense mutation c.1459G > A p.Glu487Lys, were dizygotic twins. Although clinical manifestations were typical for SEI, the phenotype was peculiar and more severe in patient *n*. 23, who presented linear hyperkeratosis in the folds, and adherent hyperkeratosis on the elbows, buttocks, knees, legs, and foot dorsum (Figure 5A,B). In addition, he showed well-defined polycyclic migrating erythematous and brownish lesions on the posterior lower limbs with focal superficial erosions and thin lamellar desquamation (Figure 5A,B). The lesions started as erythematous patches rapidly enlarging and developing central erosions and peripheral desquamation. Ichthyosis features were significantly milder in the second twin (*n*. 24). Both brothers had hypertrichosis. Their father (*n*. 25) was also affected presenting only mild limb hyperkeratosis with focal superficial desquamation. Skin histopathology showed vacuolation of the granular, but also upper suprabasal epidermal layers, with numerous eosinophilic inclusions, corresponding to tonofilament clumps visualized at TEM examination (Figure 5C,D).

## 3. Discussion

Our case series confirms the significant clinical phenotype variability of *KRT1*, *KRT2*, and *KRT10* gene mutations [7,16,22,23,24]. Indeed, a wide spectrum of skin involvement extent and severity has been observed not only in different kindred, but also between siblings (*n*. 1–2 and 23–24). Disease severity mainly depends on how keratin mutations impact the molecular structure of keratin filaments. As an example, among missense mutations affecting the K1/10 rod domain, those that fall within the highly conserved helix boundary motifs, the helix initiation and termination peptides, are usually associated with more severe EI phenotypes. Recent structural biochemistry advances have also been relevant to improve genotype–phenotype correlation. In particular, the resolved crystal structure of specific keratin subdomains can assist in modelling mutations and better deciphering their effects on keratin assembly, allowing more precise genotype–structurotype–phenotype correlation [25]. On the other hand, sibling-to-sibling variability suggests that additional genetic and epigenetic factors may modify the disease course. Furthermore, examination of patients from different generations within the same family confirmed that disease severity tends to decrease with age (*n*. 1–3, 5–6, 10–11, and 23–25). As to mutations in *KRT1* and *KRT10* genes, major differences in the clinical presentation, evident from the neonatal age and maintaining lifelong, were detected in our series. Thus, the phenotype was quite mild in some patients, who had minimal (e.g., *n*. 4) or even overlooked (*n*. 1–3) skin fragility in infancy, followed by localized hyperkeratosis. At the other end of the EI phenotypic spectrum, patients presented neonatal life-threatening skin fragility and erythroderma, and then developed severe diffuse hyperkeratosis with major functional damage and growth delay (*n*. 12–14). As expected, PPK was a typical but not exclusive feature of *KRT1* mutated patients, as it was observed also in one patient with *KRT10* mutation (*n*. 14), and in patient *n*. 22 affected with SEI due to a *KRT2* in-frame deletion [15]. Indeed, PPK is uncommon in EI patients due to *KRT10* mutations [24] and usually not described in SEI. Our two patients affected with CRIE due to *KRT10* mutations also had marked and progressive PPK, in line with literature findings [8]. Moreover, patient *n*. 20 developed mental retardation as reported in other CRIE cases [16]. On the other hand, patients with SEI (*n*. 21–25) presented a mild phenotype, as expected [16,20]. Interestingly, the polycyclic migrating erythematous patches observed in one SEI case (*n*. 23) were reminiscent of lesions in annular EI. Similar features of annular erythema have been reported in a Japanese SEI case [26]. Of note, all SEI patients presented hypertrichosis; this feature has been previously described in SEI as a distinctive clinical sign useful in disease diagnosis [27,28]. However, in our series, hypertrichosis was also present in eight EI cases, five due to *KRT1* (*n*. 1, 2, 5–7) and three to *KRT10* mutations (*n*. 9, 13, 14). To our knowledge, hypertrichosis has been previously reported only in EI patients with an intermediate severity phenotype and without PPK [23]. In addition, our two patients with CRIE (*n*. 19, 20) had marked and generalized hypertrichosis, in keeping with literature findings [8,16]. Thus, hypertrichosis seems to be a frequent and underestimated feature in KI. However, a direct effect of keratin mutations on hair is not likely, as K2 is not expressed in hair follicles, and K1/K10 are completely lost in the infundibulum. Cytokines and growth factors produced during the inflammatory processes typically associated with KI might play a role in modulating the hair cycle. Itching was a frequent symptom in all KI, and together with pain, in particular in CRIE patients, affected the quality of life. De Palma et al. described itching in all ichthyosis types as the second most important symptom after pain, with physical and psychological impact on daily life [29]. 

In view of the phenotypic spectrum of KI and the similarities of histopathological findings among different KI forms, only genetic testing may allow us to define the specific disease type with relevant implications for prognostication and care, in addition to genetic counselling. Indeed, several patients in our series (e.g., 1–4, 19, 20) were correctly diagnosed only following genetic testing, which allowed us to identify the pathogenic mutations in all the 25 cases analyzed. Moreover, the mother (*n*. 15) of patient *n*. 14 received the diagnosis of EN only following the diagnosis of EI in her son. This observation further highlights the need for skin biopsies for histopathology and genetic testing in patients with extensive epidermal nevus, in order to early diagnose EN and provide proper counselling on the risk of disease transmission to the offspring [10,11,12,13]. To this end, here we provide additional evidence that massive parallel sequencing inherent to NGS technology can help in identifying mutant alleles present at low frequency in the genomic DNA purified from the entire biopsy of lesional EN skin, and even from blood, in particular when the post-zygotic mutational event has occurred early in the embryo. Importantly, NGS identified for the first time a somatic *KRT2* mutation, p.Asn186Asp, in a patient presenting with an extensive epidermal nevus. In patients with epidermal nevi, the presence of focal vacuolar degeneration within the granular and spinous epidermal layers is distinctive for EN. These findings were present also in our patient. Thus, histopathological features appear indistinguishable between “classical” EN due to *KRT1/10* mutations and EN associated with *KRT2* somatic mutation. This suggests that mosaicism in *KRT2* may underlie a subset of EN, as EN diagnosis is usually based on histopathology findings only. Regular genetic testing of EN patients could therefore unveil additional cases due to *KRT2* somatic mutations. Similar to *KRT10*-mutated EN, it may be hypothesized that *KRT2* EN may also be transmitted as SEI to the offspring when somatic mutations involve germline cells. However, the risk of transmission, if any, remains to be determined. 

Finally, we report on three novel mutations which contribute to expand the keratin mutation database and confirm that both the position of the mutation within K1, K10, and K2 and the nature of amino acid substitution influence phenotype. However, our findings also further highlight the complexity of genotype–phenotype correlation in KI. The differences in genotype–phenotype correlations may be not only age-related, but also dependent on other genetic and epigenetic factors that remain to be identified.

## 4. Materials and Methods 

### 4.1. Study Population

The series included KI patients seen at the Reference Center for Rare Skin Diseases, Dermatology Unit, Bambino Gesù Children’s Hospital between 2010 and 2019. Local Ethics Committee approval was obtained (*n*. 2251). Family history, and clinical and follow-up information were extracted from hospital electronic records. According to our diagnostic ichthyosis protocol, patients underwent at first skin biopsies for histopathological and TEM examination, if not already performed. Blood sampling, and in two cases skin biopsies, were then obtained for genetic diagnosis in affected patients and their parents. Genetic analysis was directly performed in 3 familial cases (*n*. 1–3 in Table 1) who came to our observation during the annual meeting of the Italian ichthyosis patients’ association (UNITI).

### 4.2. Molecular Genetic Diagnosis

Following informed consent, patient genomic DNA was extracted from peripheral blood and, in two cases with EN (*n*. 17 and 26 in Table 1), also from the whole biopsy obtained from lesional skin. Mutations were identified through the targeted next generation sequencing (NGS) approach using a customized ichthyosis gene panel (Nextera Rapid Capture Custom Enrichment Kit, Illumina, San Diego, CA, USA; NimbleGen SeqCap Target Enrichment, Roche, Madison, WI, USA) and MiSeq or NextSeq550 sequencing platforms (Illumina). Identified variants were evaluated by the web-based tool VarSome [30] and validated by Sanger sequencing.

### 4.3. Ethics Statement

This research was approved by the Ethics Committee of the Bambino Gesù Children’s Hospital (2251, 1th Oct 2020). Written informed consent was obtained from each of the donors.

## Figures and Tables

**Table 1:** Clinical and molecular features of 26 keratinopathic ichthyosis patients.

Pt N *	Sex	Age °	Family History	Gene and Mutation (cDNA and Protein)	Diagnosis	Skin Phenotype	PPK ”	Hypertrichosis	Hypohidrosis	Itching	Growth Delay
01	M **	21 yrs.	Yes (mother pt 3, sister pt 2)	°° KRT1: c.1792dupA; p.Ser598Lysfs*56	EI ^§^	Onset at birth; focal hyperkeratosis marked at joints; face erythema	Yes	Yes	No	No	No
02	F ***	19 yrs.	Yes (mother pt 3, brother pt 1)	KRT1: c.1792dupA; p.Ser598Lysfs*56	EI	Onset at birth; focal hyperkeratosis marked at joints; minimal face erythema	Yes	Yes	No	No	No
03	F	53 yrs.	Yes (daughter pt 2, son pt 1)	KRT1: c.1792dupA; p.Ser598Lysfs*56	EI	Onset at 6 mo °°°; focal hyperkeratosis	Yes	No	No	No	No
04	F	7 yrs.	Yes (father)	**KRT1: c.1319C > T; p.Ala440Val**	EI	First mo: erosions and desquamation; then progressive focal hyperkeratosis	Yes	No	No	Yes	No
05	M	7 mo	Yes (mother pt 6)	KRT1: c.531G > T; p.Lys177Asn	EI	Birth: erythroderma, superficial blisters, and desquamation; then localized mild hyperkeratosis and few erosions	Yes	Yes	No	No	No
06	F	32 yrs.	Yes (son pt 5)	KRT1: c.531G > T; p.Lys177Asn	EI	Birth: erythroderma, superficial blisters, and desquamation; then localized mild hyperkeratosis	Yes	Yes	Yes	No	No
07	M	34 yrs.	Negative	KRT1: c. 563A > G; p.Asn188Ser	EI	Birth: erythroderma, superficial blisters, and desquamation; then erythema, adherent hyperkeratosis, and erosions	Yes	Yes	NK ^	Yes	No
08	F	9 yrs.	Negative	KRT10: c.467G > A; p.Arg156His	EI	Birth: erythroderma, blisters, erosions; then diffuse hyperkeratosis marked at joints	No	No	Yes	Yes	No
09	F	30 yrs.	Negative	KRT10: c.466C > T; p.Arg156Cys	EI	Birth: erythroderma, blisters, erosions; then diffuse hyperkeratosis marked at joints	No	Yes	Yes	No	No
10	F	6 mo	Yes (mother pt 11)	KRT10: c.1346A > G; p.Tyr449Cys	EI	Two days: blisters, erosions, and mild erythema; infancy lamellar desquamation	No	No	NK	NK	No
11	F	31 yrs.	Yes (daughter pt 10)	KRT10: c.1346A > G; p.Tyr449Cys	EI	First month: blisters, erosions and mild erythema; adulthood: mild focal hyperkeratosis (joints)	No	No	NK	No	No
12	M	1 mo	Negative	KRT10: c.466C > T; p.Arg156Cys	EI	Birth: erythroderma, blisters, erosions; then marked, adherent, and diffuse hyperkeratosis, erosions and erythema	No	No	NK	Yes	Yes
13	M	3 mo	Negative	**KRT10: c.461A > G; p.Asn154Ser**	EI	Birth: erythroderma, blisters, erosions; then marked, adherent, and diffuse hyperkeratosis, erosions and erythema	Yes	Yes	NK	Yes	Yes
14	M	1 mo	Yes (mother pt 15)	KRT10: c.466C > T; p.Arg156Cys	EI	Birth: erythroderma, blisters, erosions; then marked, adherent, and diffuse hyperkeratosis, erosions and erythema	No	Yes	Yes	Yes	Yes
15	F	42 yrs.	Yes (son pt 14)	KRT10: c.466C > T; p.Arg156Cys	EN ^§§^	Birth: blaschkoid hyperkeratosis and erosions	No	No	Yes	Yes	No
16	F	40 yrs.	Negative	KRT10: c.467G > A; p.Arg156His	EI	Birth: erythroderma, blisters, erosions; then adherent and diffuse hyperkeratosis, erosions more evident on the limbs	No	NK	NK	Yes	No
17	F	7 yrs.	Negative	KRT10: c.466C > T; p.Arg156Cys (on skin: 18%)	EN	2 mo: monolateral blaschkoid hyperkeratosis, hypopigmentation, and erosions	No	No	No	No	No
18	F	4 mo	Negative	Not performed	EI	Birth: erythroderma, blisters, erosions; infancy: diffuse desquamation, hyperkeratosis, erosions, and erythema	Yes	No	NK	Yes	Yes
19	M	2 mo	Negative	KRT10: c.1374-1G > C; p.Ser458Argfs*120	CRIE ^§§§^	Birth: collodion baby; then: ichthyosiform erythroderma; normal skin areas from 5 years	Yes	Yes	Yes	Yes	Yes
20	F	2 mo	Negative	KRT10: c.1383_1414del32; p.Gly462Leufs*108	CRIE	Birth: collodion baby; then: ichthyosiform erythroderma; normal skin areas from 2.5 years; psychomotor delay	Yes	Yes	Yes	Yes	yes
21	M	5 yrs.	Negative	KRT2: c.1459G > A; p.Glu487Lys	SEI ^§§§§^	4 mo: blisters, erosions and superficial desquamation; then mild localized erythema, hyperkeratosis mainly at buttocks and limbs	No	Yes	Yes	Yes	No
22	F	27 yrs.	Yes (mother)	KRT2: c.561_563delCAA; p.Asn187del	SEI	2 mo: erosions and superficial desquamation; then hyperkeratosis mainly at limbs	Yes (focal)	Yes	No	Yes	No
23	M	7 yrs.	Yes (brother pt 24, father pt 25)	KRT2: c.1459G > A; p.Glu487Lys	SEI	18 mo: erosions and superficial desquamation; then hyperkeratosis on lower limbs, elbows and buttocks, migrating erythematous brownish patches and superficial desquamation	No	Yes	No	Yes	No
24	M	7 yrs.	Yes (brother pt 23, father pt 25)	KRT2: c.1459G > A; p.Glu487Lys	SEI	18 mo: erosions and superficial desquamation; then mild and localized hyperkeratosis on feet, knees, and elbows, with focal superficial desquamation	No	Yes	No	Yes	No
25	M	43 yrs.	Yes (both sons, pts 23 and 24)	KRT2: c.1459G > A; p.Glu487Lys	SEI	Childhood: erosions and superficial desquamation; then focal hyperkeratosis on limbs and superficial desquamation	No	Yes	No	Yes	No
26	M	3 yrs.	Negative	KRT2: c.556A > G; p.Asn186Asp	EN	6 mo: blaschkoid hypochromic streaks on the lower limbs, then blaschkoid brownish hyperkeratosis on the inguinal and infragluteal folds, and less marked on axillae, elbows, knees, and foot dorsum	No	No	No	No	No

* Pt N: patient number; ** M: male; *** F: female; ° Age at referral to our center; ” PPK: palmoplantar keratoderma; °° novel mutations are in bold and underlined; °°° mo: month; ^ NK: Not known; § EI: epidermolytic ichthyosis; §§ EN: epidermolytic nevus; §§§ CRIE: congenital reticular ichthyosiform erythroderma; §§§§ SEI: superficial epidermolytic ichthyosis.

## Supplementary Materials

Supplementary materials can be found at https://www.mdpi.com/1422-0067/21/20/7707/s1.

## Abbreviations

CRIE	Congenital reticular ichthyosiform erythroderma
EI	Epidermolytic ichthyosis
EN	Epidermolytic nevus
K1	Keratin 1
K2	Keratin 2
K10	Keratin 10
KI	Keratinopathic ichthyoses
NGS	Next generation sequencing
SEI	Superficial epidermolytic ichthyosis
TEM	Transmission electron microscopy

## References

[1] Oji V., Tadini G., Akiyama M., Bardon C.B., Bodemer C., Bourrat E., Coudiere P., DiGiovanna J.J., Elias P., Fischer J. (2010). Revised nomenclature and classification of inherited ichthyoses: Results of the First Ichthyosis Consensus Conference in Sorèze 2009. J. Am. Acad. Derm..

[2] Has C., Bauer J.W., Bodemer C., Bolling M.C., Bruckner-Tuderman L., Diem A., Fine J.D., Heagerty A., Hovnanian A., Marinkovich M.P. (2020). Consensus reclassification of inherited epidermolysis bullosa and other disorders with skin fragility. Br. J. Derm..

[3] Vahlquist A., Fischer J., Törmä H. (2018). Inherited nonsyndromic ichthyoses: An update on pathophysiology, diagnosis and treatment. Am. J. Clin. Derm..

[4] Müller F.B., Huber M., Kinaciyan T., Hausser I., Schaffrath C., Krieg T., Hohl D., Korge B.P., Arin M.J. (2006). A human keratin 10 knockout causes recessive epidermolytic hyperkeratosis. Hum. Mol. Genet..

[5] Covaciu C., Castori M., De Luca N., Ghirri P., Nannipieri A., Ragone G., Zambruno G., Castiglia D. (2010). Lethal autosomal recessive epidermolytic ichthyosis due to a novel donor splice-site mutation in KRT10. Br. J. Derm..

[6] Tsubota A., Akiyama M., Kanitakis J., Sakai K., Nomura T., Claudy A., Shimizu H. (2008). Mild recessive bullous congenital ichthyosiform erythroderma due to a previously unidentified homozygous keratin 10 nonsense mutation. J. Investig. Derm..

[7] Arin M.J., Oji V., Emmert S., Hausser I., Traupe H., Krieg T., Grimberg G. (2011). Expanding the keratin mutation database: Novel and recurrent mutations and genotype-phenotype correlations in 28 patients with epidermolytic ichthyosis. Br. J. Derm..

[8] Guerra L., Diociaiuti A., El Hachem M., Castiglia D., Zambruno G. (2015). Ichthyosis with confetti: Clinics, molecular genetics and management. Orphanet J. Rare Dis..

[9] Nazzaro V., Ermacora E., Santucci B., Caputo R. (1990). Epidermolytic hyperkeratosis: Generalized form in children from parents with systematized linear form. Br. J. Derm..

[10] Paller A.S., Syder A.J., Chan Y.M., Yu Q.C., Hutton E., Tadini G., Fuchs E. (1994). Genetic and clinical mosaicism in a type of epidermal nevus. N. Engl. J. Med..

[11] Chassaing N., Kanitakis J., Sportich S., Cordier-Alex M.P., Titeux M., Calvas P., Claudy A., Berbis P., Hovnanian A. (2006). Generalized epidermolytic hyperkeratosis in two unrelated children from parents with localized linear form, and prenatal diagnosis. J. Investig. Derm..

[12] Tsubota A., Akiyama M., Sakai K., Goto M., Nomura Y., Ando S., Abe M., Sawamura D., Shimizu H. (2007). Keratin 1 gene mutation detected in epidermal nevus with epidermolytic hyperkeratosis. J. Investig. Derm..

[13] Kono M., Suga Y., Akashi T., Ito Y., Takeichi T., Muro Y., Akiyama M. (2017). A Child with epidermolytic ichthyosis from a parent with epidermolytic nevus: Risk evaluation of transmission from mosaic to germline. J. Investig. Derm..

[14] Ross R., DiGiovanna J.J., Capaldi L., Argenyi Z., Fleckman P., Robinson-Bostom L. (2008). Histopathologic characterization of epidermolytic hyperkeratosis: A systematic review of histology from the national registry for ichthyosis and related skin disorders. J. Am. Acad. Derm..

[15] Diociaiuti A., El Hachem M., Pisaneschi E., Giancristoforo S., Genovese S., Sirleto P., Boldrini R., Angioni A. (2016). Role of molecular testing in the multidisciplinary diagnostic approach of ichthyosis. Orphanet J. Rare Dis..

[16] Hotz A., Oji V., Bourrat E., Jonca N., Mazereeuw-Hautier J., Betz R.C., Blume-Peytavi U., Stieler K., Morice-Picard F., Schönbuchner I. (2016). Expanding the clinical and genetic spectrum of KRT1, KRT2 and KRT10 mutations in keratinopathic ichthyosis. Acta Derm. Venereol..

[17] Sprecher E., Yosipovitch G., Bergman R., Ciubutaro D., Indelman M., Pfendner E., Goh L.C., Miller C.J., Uitto J., Richard G. (2003). Epidermolytic hyperkeratosis and epidermolysis bullosa simplex caused by frameshift mutations altering the v2 tail domains of keratin 1 and keratin 5. J. Investig. Derm..

[18] Liu X.P., Ling J., Xiong H., Shi X.L., Sun X., Pan Q., Hu Z.M., Wu L.Q., Liang D.S., Long Z.G. (2009). Mutation L437P in the 2B domain of keratin 1 causes diffuse palmoplantar keratoderma in a Chinese pedigree. J. Eur. Acad. Derm. Venereol..

[19] Chipev C.C., Yang J.M., DiGiovanna J.J., Steinert P.M., Marekov L., Compton J.G., Bale S.J. (1994). Preferential sites in keratin 10 that are mutated in epidermolytic hyperkeratosis. Am. J. Hum. Genet..

[20] Takizawa Y., Akiyama M., Nagashima M., Shimizu H. (2000). A novel asparagine-->aspartic acid mutation in the rod 1A domain in keratin 2e in a Japanese family with ichthyosis bullosa of Siemens. J. Investig. Derm..

[21] Diociaiuti A., Fortugno P., El Hachem M., Angelo C., Proto V., De Luca N., Martinelli D., Boldrini R., Castiglia D., Zambruno G. (2014). Early immunopathological diagnosis of ichthyosis with confetti in two sporadic cases with new mutations in keratin 10. Acta Derm. Venereol..

[22] Virtanen M., Smith S.K., Gedde-Dahl T., Vahlquist A., Bowden P.E. (2003). Splice site and deletion mutations in keratin (KRT1 and KRT10) genes: Unusual phenotypic alterations in Scandinavian patients with epidermolytic hyperkeratosis. J. Investig. Derm..

[23] DiGiovanna J.J., Bale S.J. (1994). Clinical heterogeneity in epidermolytic hyperkeratosis. Arch. Derm..

[24] Bygum A., Virtanen M., Brandrup F., Gånemo A., Sommerlund M., Strauss G., Vahlquist A. (2013). Generalized and naevoid epidermolytic ichthyosis in Denmark: Clinical and mutational findings. Acta Derm. Venereol..

[25] Hinbest A.J., Eldirany S.A., Ho M., Bunick C.G. (2020). Molecular modeling of pathogenic mutations in the keratin 1B domain. Int. J. Mol. Sci..

[26] Akiyama M., Tsuji-Abe Y., Yanagihara M., Nakajima K., Kodama H., Yaosaka M., Abe M., Sawamura D., Shimizu H. (2005). Ichthyosis bullosa of Siemens: Its correct diagnosis facilitated by molecular genetic testing. Br. J. Derm..

[27] Basarab T., Smith F.J., Jolliffe V.M., McLean W.H., Neill S., Rustin M.H., Eady R.A. (1999). Ichthyosis bullosa of Siemens: Report of a family with evidence of a keratin 2e mutation, and a review of the literature. Br. J. Derm..

[28] Gameiro A., Cabral R., Moreno A., Tellechea O. (2016). Superficial epidermolytic ichthyosis-Hypertrichosis as a clue to diagnosis. Pediatr. Derm..

[29] De Palma A.M., Mazereeuw-Hautier J., Giehl K., Hernández-Martin A., Merlos M., Moons P., Morren M.A. (2019). Burden of itch in ichthyosis: A multicentre study in 94 patients. J. Eur. Acad. Derm. Venereol..

[30] Kopanos C., Tsiolkas V., Kouris A., Chapple C.E., Albarca Aguilera M., Meyer R., Massouras A. (2019). VarSome: The human genomic variant search engine. Bioinformatics.

