# Supplementary material for: First Case of KRT2 Epidermolytic Nevus and Novel Clinical and Genetic Findings in 26 Italian Patients with Keratinopathic Ichthyoses
Source: Int J Mol Sci. 2020 Oct 18;21(20):7707. doi: 10.3390/ijms21207707 (PMC7593923; doi:10.3390/ijms21207707)
Supplement: Supplementary file 1 [file ijms-21-07707-s001.pdf]

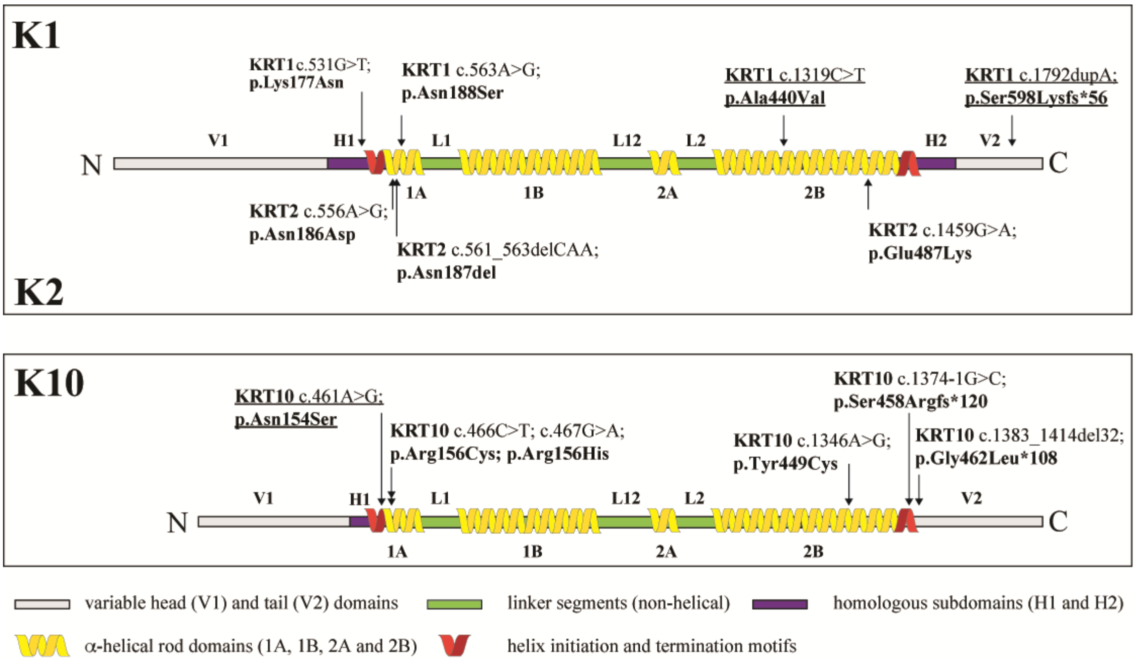

Figure S1. Keratin 1 (K1), K2 and K10 structural organization with the position of the pathogenic variants identified in this study. Novel mutations are underlined.

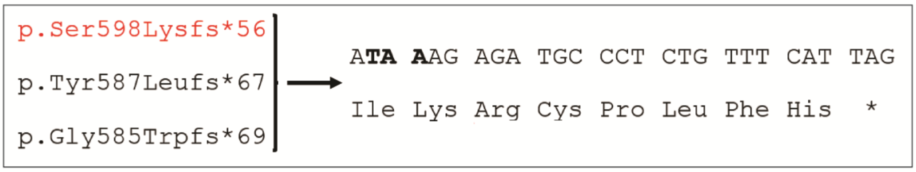

Figure S2. *KRT1* frameshift mutations (listed on the left) that lead to stop codon readthrough resulting in an identical extension of 7 amino acids after the readthrough (right). The novel mutation described in this study is depicted in red; the natural stop codon is in bold.
